# Balneotherapy, Immune System, and Stress Response: A Hormetic Strategy?

**DOI:** 10.3390/ijms19061687

**Published:** 2018-06-06

**Authors:** Isabel Gálvez, Silvia Torres-Piles, Eduardo Ortega-Rincón

**Affiliations:** 1 Research Group in Immunophysiology, Department of Physiology, Faculty of Sciences, University of Extremadura, Avda. Elvas s/n, 06071 Badajoz, Spain; igalvez@unex.es; 2 Research Group in Immunophysiology, Department of Medical-Surgical Therapy, Faculty of Medicine, University of Extremadura, Avda. Elvas s/n, 06071 Badajoz, Spain; storres@unex.es

**Keywords:** spa therapy, mud therapy, hydrotherapy, hormesis, immune response, inflammation, oxidative stress, heat shock proteins, pain, rheumatic diseases

## Abstract

Balneotherapy is a clinically effective complementary approach in the treatment of low-grade inflammation- and stress-related pathologies. The biological mechanisms by which immersion in mineral-medicinal water and the application of mud alleviate symptoms of several pathologies are still not completely understood, but it is known that neuroendocrine and immunological responses—including both humoral and cell-mediated immunity—to balneotherapy are involved in these mechanisms of effectiveness; leading to anti-inflammatory, analgesic, antioxidant, chondroprotective, and anabolic effects together with neuroendocrine-immune regulation in different conditions. Hormesis can play a critical role in all these biological effects and mechanisms of effectiveness. The hormetic effects of balneotherapy can be related to non-specific factors such as heat—which induces the heat shock response, and therefore the synthesis and release of heat shock proteins—and also to specific biochemical components such as hydrogen sulfide (H_2_S) in sulfurous water and radon in radioactive water. Results from several investigations suggest that the beneficial effects of balneotherapy and hydrotherapy are consistent with the concept of hormesis, and thus support a role for hormesis in hydrothermal treatments.

## 1. Introduction

Hormesis is a biphasic dose-response phenomenon in which exposure of a cell or organism to a low dose of a chemical agent or condition induces stimulation or adaptive beneficial effects, while higher doses cause inhibition or toxic effects [1,2]. This response to low doses of stress is considered an adaptive compensatory process or adaptive stress response following an initial disruption in homeostasis, enhancing the ability of the organism to withstand more severe stress [1,2]. Therefore, a hormetin has been defined as any condition that may be potentially hormetic in physiological terms by activating or upregulating one or more cellular and molecular pathways of stress response that protect against a similar but more severe stress [3]. Apart from chemicals and toxins, there are several conditions and factors that can be considered hormetins: biological hormetins such as infections, hypoxia/ischemia, endogenous metabolic products, dietary caloric restriction, intermittent fasting, and micronutrients; psychological hormetins such as mental challenge and meditation; and physical hormetins such as exercise, heat, and radiation [3,4]. Indeed, repeated mild heat stress-induced hormesis affects various parameters of cellular aging and other functional characteristics, such as differentiation, wound healing and angiogenesis. These hormetic effects lead to a significant biological response that results in an overall improvement of the living system [5]. In this context, thermotherapeutic strategies such as balneotherapy and hydrotherapy can be considered potential hormetic interventions.

In Medical Hydrology and Physical Medicine, spa therapy consists of multiple techniques based on the healing effects of water, including balneotherapy and hydrotherapy. Balneotherapy is the set of methods and practices which, based on scientific evidence, use medically and legally recognized mineral-medicinal waters, muds, and natural gases from natural springs for therapeutic purposes inside the facilities of thermal spa centres. Particularly, muds (or peloids) are maturated muddy suspensions composed of a complex mixture of fine-grained materials of geologic origin, mineral water, and commonly organic compounds from biological metabolic activity. Thus, mud therapy or pelotherapy is a balneological intervention that consists of the external application of mud for therapeutic purposes [6]. Temperature has a central role in the effects of balneotherapy. Mineral-medicinal water and mud are generally applied hot since they are excellent vehicles for the transference of heat—being able to hold heat and release it slowly—so these treatments can be considered thermotherapeutic interventions. The peculiarity of balneotherapy is that its beneficial effects on the organism are brought about not only by the physical properties of mineral-medicinal water and mud, but also by their chemical and biological composition. Conversely, in other spa treatments such as hydrotherapy—in which plain tap water is used—only the physical properties of water (temperature, hydrostatic pressure, hydrodynamics, buoyancy, viscosity, electric conductivity, etc.) take part in the beneficial effects of the intervention [6].

In the last years, there has been an increase in the number of investigations related to the biological effects and mechanisms of effectiveness of these treatments, in which hormesis could play a critical role. In this review we will summarize the current knowledge about the clinical benefits and physiological effects of balneotherapy on the immune and stress response, and most importantly, we will discuss the recent progress made in the study of the hormetic mechanisms of hydrothermal treatments, focusing on balneotherapy and its different modalities.

## 2. Balneotherapy as a Strategy for Health

Balneotherapy and mud therapy have been used empirically since time immemorial to treat a wide range of conditions [7]. Thermal baths are considered an integral part of traditional medicine in many cultures and countries (France, Italy, Spain, Portugal, Germany, Austria, Switzerland, Turkey, Poland, Czech Republic, Hungary, Romania, Russia, Israel, Japan and others), and nowadays they are a relevant part of the public health systems of many countries within and outside Europe [8].

Balneotherapy is an effective, well tolerated, complementary approach in the treatment of several pathologies—mainly those related to chronic inflammation—such as cardiovascular, respiratory, gastrointestinal, endocrine, and neurological conditions, and more importantly in skin and rheumatic disorders [9,10]. In the recent decades, more and more studies (including high-quality meta-analysis and systematic reviews) have reported the beneficial effects of balneotherapy, including mud therapy, on different clinical outcomes in patients with osteoarthritis (OA) [11,12,13,14,15,16], rheumatoid arthritis (RA) [17,18,19], fibromyalgia (FM) [20,21,22,23], and other rheumatic conditions [24]. Of all these pathologies, OA is the most commonly treated with balneological interventions. The main clinical parameters improved by balneotherapy and mud therapy in OA are analgesic drug consumption, function, stiffness, pain, and quality of life [13,14]. Since these therapies have little to no adverse effects, they are especially important for OA patients, who usually are elderly patients with multimorbidity- and polypharmacy-related risk of adverse events. In fact, the most recent guidelines from the Osteoartritis Research Society International (OARSI) state that balneotherapy is appropriate in OA patients with comorbidities, for whom treatment options are limited [25].

Balneotherapy causes local and generalized physiological effects in the organism, which are exerted through both physical mechanisms—mainly linked to heat therapeutic effects—and chemical and biological properties of the agents [9]. While the former are well known [26], the latter are difficult to identify and assess [27]. Indeed, as a result of the elevated application temperature—generally ranging from 38 to 42 °C—thermotherapeutic effects are the basis of these treatments [27,28]. Notwithstanding, absorption of biologically active inorganic and organic substances through the skin also play a role in the effectiveness of balneotherapy. In vitro and in vivo studies have established that some water-soluble minerals are able to permeate human skin [29,30,31] and seem to be the key mechanism responsible for the improvement in some clinical outcomes, in both balneotherapy and mud therapy [30,32,33,34,35,36,37,38,39], thus implying that those beneficial effects are not exclusively linked to the action of heat. Despite this increasing evidence, it is difficult to analyze the specific effects of each mechanism and each chemical component separately. Each mineral-medicinal water and mud around the world has different distinctive physical properties and chemical composition. According to their predominant ions and gases, mineral-medicinal waters may be classified as chlorated, sulfated, bicarbonated, ferruginous, carbogaseous, sulfurous and radioactive [40]. It is known that, generally, different diseases require agents with different chemical compositions in order to attain therapeutic results [41]. However, the exact components that are most suitable for each pathology and the ideal concentration of each element that is necessary for obtaining optimal biological and clinical outcomes have not yet been completely elucidated.

It is plausible to think that the mechanism of action probably results from a complex synergistic combination of several factors [27,39]. Whichever of these mechanisms are implicated to a lesser or larger extent, the physiological responses arising from balneotherapy consist mainly of neuroendocrine and immunological effects that have been most widely studied in rheumatic pathologies.

## 3. Balneotherapy and Immune System

The biological mechanisms by which immersion in mineral-medicinal water and the application of mud alleviate symptoms of several pathologies are still not completely understood. For the last two decades, immunological mechanisms of effectiveness have been studied in a number of investigations, pointing to anti-inflammatory effects that could underlie the clinical benefits of balneotherapy.

In several low-grade inflammation-related pathologies—especially in rheumatic diseases—balneotherapy and mud therapy have been reported to cause a reduction in serum concentrations of pro-inflammatory cytokines TNF-α [42,43,44,45,46] and IL-1β [43,47], and regulatory cytokine IL-6 [46,48], as well as an increase in anti-inflammatory growth factor IGF-1 [38,42]. It is noteworthy that, in a recent study, our group carried out a comprehensive evaluation of the effect of mud therapy on the cytokine profile of OA patients. Our data showed a drastic decline in the unhealthily elevated systemic levels of IL-1β, TNF-α, IL-8 (remarkably for the first time), IL-6 and TGF-β, thus confirming a global anti-inflammatory effect of this strategy [49]. Mud therapy can also decrease circulating levels of the adipokines adiponectin and resistin in OA [50,51]. All these cytokines and adipokines are important mediators of inflammation and cartilage metabolism [52], and thus their modulation after balneotherapy leads to anti-inflammatory-mediated chondroprotective effects that may play a beneficial role in rheumatic conditions such as OA. 

Similarly, matrix metalloproteinases (MMP) are involved in cartilage degradation [53]. MMP-3 serum levels decrease after mud therapy in OA patients [54]—either as a direct effect of the intervention or as a consequence of the reduction in pro-inflammatory mediators such as cytokines that promote MMP secretion—suggesting that mud therapy contributes to extracellular matrix integrity. In fact, serum cartilage oligomeric matrix protein (COMP) concentration—an indicator of cartilage turnover—decreases after balneotherapy [45]. 

Moreover, C-reactive protein (CRP) levels—which rise in response to inflammation—decrease after balneotherapy in patients with rheumatic and cardiovascular pathologies [46,55,56]. Prostaglandin E2 (PGE2) and leukotriene B4 (LTB4) are other important inflammatory mediators [57]. There is evidence that balneotherapy and mud therapy reduce circulating levels of these biomarkers in patients with rheumatic pathologies [47,58]. It is well known that the inflammatory response plays a key role in the development and persistence of many pathological pain states. Since they are part of the inflammatory response, certain pro-inflammatory cytokines such as IL-1β, IL-6, and TNF-α are strongly involved in the process of inflammatory and neuropathic pain. They can directly modulate neuronal activity in the peripheral and central nervous system and promote the production of other mediators related to inflammation and pain—such as substance P and PGE2—contributing to hyperalgesia and allodyinia [59]. In this way, reduction in these mediators’ levels after balneotherapy can also be associated with the analgesic effects of the treatment, as demonstrated by concomitant declines in perceived pain [47,49].

Cellular immune response also participates in the beneficial effects of balneotherapy, although not many studies have been conducted regarding this aspect of the immune response. Recently, our research group has found that OA patients presented a reduction in the circulating neutrophils’ functional capacity—i.e., phagocytic and microbicidal activities [60]—that improved significantly after mud therapy. Circulating monocytes’ phenotype and functional capacity seem to be also involved in the innate/inflammatory response induced by this treatment. In addition, changes in the percentage of circulating regulatory T cells are also implicated in the cytokine-mediated anti-inflammatory effect of balneotherapy (unpublished data, submitted for publication).

## 4. Balneotherapy and Stress

It is known that the hypothalamic-pituitary-adrenal (HPA) axis is activated in response to various stress factors—including hyperthermia—leading to β-endorphin (a peptide with morphine-like analgesic effects [61]), adrenocorticotropic hormone (ACTH), and cortisol release [62], the latter being especially important because of its anti-inflammatory effects and ability to inhibit the production of most cytokines [63]. At the same time, activation of the sympathetic nervous system (SNS) by stressors stimulates the release of catecholamines [62].

Hyperthermia-induced activation of the HPA axis and SNS has been reported mostly in healthy subjects undergoing plain hydrotherapy or sauna baths [64,65]. This activation was manifested by increased circulating concentrations of ACTH [66,67], cortisol [66,68], growth hormone (GH) [68,69], prolactin [70,71], β-endorphins [66,71,72], and noradrenaline (NA) [67,69,70,73]. However, there is scarce evidence on the specific effects of balneotherapy on the neuroendocrine/stress response. Besides, it is important to study these effects in different pathologies and conditions, since the presence of HPA axis and neuroendocrine-immune dysregulations in rheumatic disorders and chronic low-grade inflammatory pathologies is very common [60,63,74].

After balneotherapy, ACTH, cortisol, GH, and prolactin systemic levels increase in patients with different pathologies, including inflammatory ones [75]. In FM patients, for example, mud therapy induces an increase in ACTH, cortisol, and β-endorphin systemic concentrations [76]. Recently, our group reported a neuroendocrine-immune regulation in OA patients undergoing mud therapy: an increase in circulating cortisol concentrations that contributes to decrease the elevated systemic levels of inflammatory cytokines in this pathology [49]. These results seem to be in line with the above-mentioned studies in hydrotherapy, thus suggesting that the effects of balneotherapy on the neuroendocrine system are mainly due to heat stress caused by the elevated temperature of application.

In addition, heat stress induces a cellular response, the heat shock (HS) response, in which heat shock proteins (Hsp) are synthesized and released. Maintenance of the HS response by repeated mild heat stress causes hormetic effects in the organism [77]. In this way, HS response and Hsp could play a role in the beneficial effects of balneotherapy [78].

**Figure 1:**
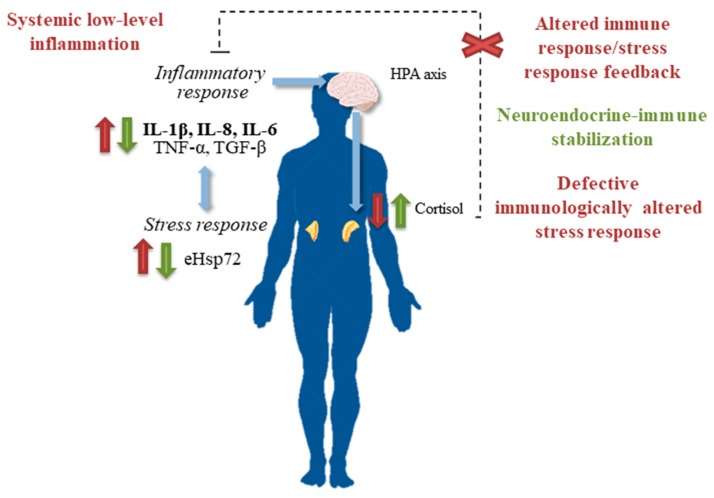
Neuroendocrine-immune stabilization as a proposed mechanism of effectiveness of a cycle of balneotherapy underlying pain alleviation in osteoarthritis (OA) patients [49]. Elevated systemic concentrations of pro-inflammatory cytokines are unable to activate cortisol release in OA patients, and the low concentration of cortisol cannot inhibit the high systemic levels of pro-inflammatory cytokines [60] (symbols and text in red). Balneotherapy increases systemic cortisol levels that in turn induce an anti-inflammatory response that is manifested by a decrease in the concentration of circulating pro-inflammatory cytokines (symbols and text in green). Balneotherapy decreased the unhealthily elevated eHsp72 concentrations in OA patients, also contributing to the anti-inflammatory effects [49]. Up and down arrows represent increases and decreases in the systemic concentrations of cytokines, eHsp72, and cortisol.

Figure 1 shows a proposed model of a mechanism of action of balneotherapy in OA patients integrating its effects on the immune and stress responses.

## 5. Balneotherapy as a Hormetic Strategy

The hormetic effects of balneotherapy are related to different factors. The main factor that is common to all types of mineral-medicinal waters and muds is heat. Other factors are specific biochemical components of water such as hydrogen sulfide (H_2_S) and radon.

### 5.1. Heat Stress Hormetic Effects in Balneotherapy

The therapeutic capacity of heat consists of changes in body tissue temperature for a certain time with the aim of producing physiological responses that contribute to support healing processes or alleviate pain and other symptoms [26,64], and it is linked to the ability of organisms to respond to stress and produce cellular responses of adaptation [3]. Whereas severe heat stress leads to cellular damage and cell death, mild heat stress induces the HS response, which protects cells and organisms from severe damage, allows resumption of normal cellular and physiological activities, and leads to a higher level of thermotolerance [79]. An important aspect of stress responses is that they have the potential to induce higher levels of stress tolerance and greater resistance to subsequent stress damage from more than one type of stress. In this way, mild heat stress can protect from oxidative stress or toxin damage [2,80]. In the HS response, cells activate a signaling pathway leading to the expression of Hsp. The Hsp70 (70 kDa heat shock protein) family consists of a class of Hsp that includes the stress-inducible Hsp70 (Hsp72, 72 kDa). Under normal physiological conditions, Hsp72 is expressed at low levels. However, following stress stimuli such as heat and inflammation, synthesis of intracellular Hsp72 (iHsp72) and release of extracellular Hsp72 (eHsp72) increase markedly. iHsp72 plays a crucial role in cytoprotection and cytotoxicity tolerance as an intracellular molecular chaperone involved in cell aging, survival, and protection against potentially harmful stress stimuli [81,82,83]. 

Of the few studies on Hsp and spa therapy that exist, most of them have assessed hydrotherapy rather than balneotherapy. The effects of hydrothermal therapy have been compared to those of exercise [84]—a hormetin with bioregulatory effects frequently used as a therapy for different pathologies [4]—since both strategies have the potential to improve impaired insulin sensitivity and boost endothelial expression of the constitutive isoform of nitric oxide synthase, promoting vascular health [84]. In fact, hydrotherapy at 38–41 °C (18 sessions of 30 min) reduced body weight, fasting plasma glucose levels and mean glycated hemoglobin levels in patients with type 2 diabetes mellitus [85]. In rats, bathing for 15 min in hot plain water (40–42 °C) increased Hsp72 heart tissue concentration contributing to cardioprotection against ischemia injury [86], and increased Hsp72 artery expression, mediating the suppression of neointimal thickening in injured arteries [87]. Furthermore, Bathaie and co-workers [88] found that diabetic rats undergoing hydrotherapy at 42 °C (60 sessions of 30 min each) presented improvements in lipid profile, antioxidant capacity, insulin secretion and advanced glycation end (AGE) products, together with an increase in serum eHsp72 levels that may be directly related to the beneficial effects of the therapy. In young and aged insulin-resistant monkeys, hydrotherapy at 40 °C (10 sessions of 30 min) improved blood pressure, glucose values, pancreatic responses to glucose challenge and tended to normalize glucose excursions, together with significantly higher concentrations of muscle Hsp70. There were no adverse effects on organ or cardiovascular health [89]. Krause and co-workers [90] proposed that all these cardiovascular and metabolic benefits of hydrotherapy seem to be related to the induction of Hsp70 expression in response to heat stress, which enhances the phosphorylation of protein kinase B (Akt), AMP-activated protein kinase (AMPK), and endothelial nitric oxide synthase. Together, they could improve insulin signaling, body composition, endothelial dysfunction, and the low-grade inflammation found in people with diabetes [90]. These investigations support the safety and efficacy of hydrotherapy as a preventive and therapeutic strategy in patients with metabolic syndrome that are too physically impaired to perform exercise at optimal intensities.

Regarding treatments with mineral-medicinal waters and muds, no changes in Hsp60 serum levels were found either after balneotherapy or hydrotherapy at 38 °C (15 sessions of 30 min) in patients with degenerative musculoskeletal disease [55], probably because the temperature was not high enough to elicit a response. Balneotherapy and mud therapy interventions (seven sessions, temperature and duration unknown) have been shown to increase Hsp70 gene expression in healthy subjects [78]. Surprisingly, our research group recently found a reduction in systemic eHsp72 concentrations in elderly OA patients after 10 sessions of balneotherapy with mud application at 38–42 °C for 60 min, in parallel with a marked decrease in the serum concentration of pro-inflammatory cytokines. OA patients presented increased serum eHsp72 and pro-inflammatory cytokines concentrations at baseline compared to age-matched healthy controls, and they reached similar values to those of controls after the therapy [49]. Similarly, Uzunoğlu and co-workers [91] assessed the effect of balneotherapy (39–40 °C for 15 min during three weeks) on Hsp in OA patients. Serum eHsp72 concentrations initially increased after the first session, but at the end of the protocol eHsp72 systemic concentration was lower than baseline, implying that an adaptation might occur at the end of the intervention. 

This paradoxical effect is associated with the role of eHsp72 as an extracellular chaperokine [81,92]. Conversely to iHsp72 (anti-inflammatory and cytoprotective), eHsp72 can act as a pro-inflammatory mediator, producing an immune/inflammatory response involving the activation of immune effector cells and cytokine release [93], particularly inflammatory cells and pro-inflammatory cytokines with the participation of nuclear factor kappa beta (NF-κβ) [94,95]. Moreover, due to its capacity to affect the production of cytokines that in turn induce neuroendocrine responses, eHsp72 is an intrinsic component of the immune-neuro-endocrine network [96]. In this way, modulation of eHsp72 circulating concentrations after heat stress could trigger an Hsp-cytokine-HPA-cortisol anti-inflammatory feedback mechanism, leading to anti-inflammatory effects and neuroendocrine-immune regulation [49]. Therefore, it could be speculated that the paradoxical decrease of eHsp72 in our study reflects a lower release of eHsp72 after a potential heat-induced iHsp72 increase in OA tissues such as chondrocytes [49]. Thus, the ratio iHsp72/eHsp72 is crucial to evaluate the effectiveness of thermotherapy [97].

Together, these findings suggest a role for Hsp in the thermotherapeutic benefits induced by balneotherapy, which supports the relationship between hormetic pathways and hydrothermal treatments. Nevertheless, it is still necessary to determine the optimal intensity, duration, and interval of heat stimulation for clinical application, particularly in inflammation- and stress-related illnesses.

### 5.2. Hydrogen Sulfide Hormetic Effects in Balneotherapy

The active molecule in sulfurous and sulfated mineral-medicinal waters is H_2_S, a hormetin that can actively penetrate the skin. While high levels of H_2_S are extremely toxic, low levels are tolerated and have potential cytoprotective effects, with anti-inflammatory and antioxidant applications [98,99]. H_2_S has important physiological functions as an endogenous cell signaling molecule on the regulation of inflammation (through NF-κβ) and oxidative stress—acting as a reactive oxygen species (ROS) scavenger and increasing levels of superoxide dismutase (SOD) and glutathione (GSH)—among many other functions [100,101].

In vitro, several studies have demonstrated antioxidant and anti-inflammatory effects of this type of waters. Recent investigations have confirmed that sulfurous waters have direct free radical-scavenging activity, reduce ROS and reactive nitrogen species (RNS) released by human neutrophils during respiratory bursts, and protect against oxidative DNA damage, thus contributing to the therapeutic effect of these waters in inflammatory respiratory diseases [102,103,104]. Fioravanti and co-workers [105] demonstrated that sulfated thermal waters inhibit nitric oxide (NO) production and apoptosis induced by IL-1β in OA chondrocytes. Moreover, another investigation showed that sulfurous water had higher antioxidant capacity against pro-oxidant stimuli than classical reference antioxidants compounds, leading to a protective effect on DNA stability and cell viability of peripheral blood mononuclear cells (PBMC) of both Alzheimer’s disease patients and healthy controls [106]. Furthermore, there is evidence that H_2_S treatment reduces both spontaneous and IL-1β-induced secretion of IL-6, IL-8 and RANTES, as well as the expression of MMP-2 and MMP-14 in cultured fibroblast-like synoviocytes from OA patients [107]. H_2_S also blocks the production of inflammatory cytokines (IL-8, IL-1β, TNF-α, IL-6 and IL-10) and counterbalances the formation of ROS and RNS by human monocytes [108], and reduces NO, PGE2, IL-6 and MMP13 released by OA chondrocytes by downregulating genes involved in the synthesis routes of these molecules as well as NF-κβ nuclear translocation [109].

In vivo, reductions in serum levels of malondialdehyde (MDA) and carbonyls, and in SOD and catalase activity [45,110,111], have been found after balneotherapy with sulfurous water in rheumatic diseases, thus reflecting a reduction in oxidative stress that may contribute to reduce the inflammatory and catabolic status. Indeed, sulfurous waters are clinically effective in the treatment of OA and RA patients [112,113].

Apart from bathing, another lesser-known modality of balneotherapy involves drinking mineral-medicinal water, namely bicarbonated, carbogaseous, and sulfurous waters. In healthy individuals, drinking sulfurous water for two weeks caused a decrease in their circulating levels of lipid and protein oxidation products (MDA, carbonyls and advanced oxidation protein products) and an increase in their antioxidant capacity and thiol levels [114]. The combination of bathing in and drinking sulfurous water is a common practice, and it can increase plasma thiol levels and decrease circulating levels of MDA, carbonlys, MMP-2, COMP and TNF-α in OA patients [45]. These improvements in the redox status could potentially confer protection against age- and disease-related oxidative damage. In a series of very interesting studies, beneficial effects of drinking sulfurous water on diabetes and long term diabetes-associated complications have been reported. Diabetic rats drank sulfurous mineral-medicinal water for 6–7 weeks. Anti-diabetic effects of sulfurous water were evidenced by increased serum concentrations of insulin, C-peptide and IGF-1, and by a reduction in glucose and glycated hemoglobin levels, indicating a return towards normal conditions [115,116,117]. Cardiac GSH and protein thiols increased while glutathione disulfide levels decreased, thus boosting the antioxidant status. This improvement in cardiac GSH levels caused a reduction in NF-κβ as well as MMP-2, procollagen-1 and Fas-L gene expression in the left ventricle. By counteracting these pro-apoptotic and pro-fibrogenic factors, sulfurous mineral water prevented the development of fibrosis in the heart [115]. Regarding diabetic nephropathy, sulfurous water counteracted the elevation of renal thiobarbituric acid reactive substances and replenished GSH levels in diabetic rats with impaired kidney function. Improvements in renal redox balance were also reflected on improved kidney function [117]. Diabetes also impairs testicular function, and drinking sulfurous water improved the seminiferous tubule structure as well as the number of spermatogenic cells and testosterone levels in diabetic rats, probably due to an increase in testicular GSH by blocking the overexpression of apoptosis-related regulatory proteins such as Bax/Bcl-2, cytochrome *c*, caspase-9 and -3, and p53 [116].

From all these studies, it can be established that balneotherapy using waters rich in H_2_S (at low concentrations as found in natural springs) is able to exert hormetic therapeutic effects in different pathological conditions related to inflammation. Moreover, hormetic effects of thermal waters rich in sulfur could be a result of the synergistic effect of two different hormetins: H_2_S and heat.

### 5.3. Radon Hormetic Effects in Balneotherapy

Mineral-medicinal waters rich in radon are radioactive and can also be considered a therapeutic hormetic strategy. Radon spa therapy consists of the intake of radon either by inhalation or by transcutaneous absorption of radon dissolved in water, and it is applied in several inflammatory diseases such as asthma, bronchitis, psoriasis and arthritis [118]. Although ionizing radiation has been shown to be carcinogenic at high doses, at low doses it produces biologically beneficial effects by initially causing low-level molecular damage, which then leads to the activation of one or more stress response pathways and therefore induces adaptive mechanisms [3] that may prevent cancer as well as other adverse health effects [1,119]. Mechanisms of radiation-induced hormetic response include activation of DNA repair, scavenging of free radicals, elimination of damaged cells by apoptosis, synthesis of stress proteins such as Hsp, and stimulation of the immune response [119,120].

A study by Yamaoka and co-workers [121] proved that radon spa therapy was more effective than thermotherapy alone in enhancing antioxidant functions (SOD and catalase activities) and in increasing ACTH, β-endorphin, and insulin levels, among other biomarkers. These results indicate that radon in spa therapy adds further beneficial hormetic outcomes to those of thermal interventions alone, suggesting a synergistic effect of heat and radon. The same group obtained similar results in another study in OA patients undergoing radon spa therapy. There was an improvement in antioxidant and immune function together with changes in pain-associated biomarkers [122]. Conversely, another study carried out in patients with degenerative musculoskeletal disorders found no significant effects on the human endocrine system after balneotherapy with a very low radon content, suggesting that a minimum radon concentration is required in order to exert biological effects [123]. Therefore, radon spa therapy at optimal radon concentrations could be a useful complementary therapy in metabolic syndrome and rheumatic diseases such as OA.

In the context of rheumatic diseases, the anti-inflammatory mechanisms of this strategy have been demonstrated. Some of these mechanisms are a decrease in NO and ROS levels, increase in heme-oxygenase 1 and TGF-β levels, TNF-α suppression, activation of transcription factors, and enhancement of regulatory T cells. Thus, low-dose ionizing radiation exposure is able to diminish pivotal inflammatory processes associated with arthritis, by inducing a switch from a pro-inflammatory to an anti-inflammatory phenotype following the hormetic response [124,125].

Furthermore, several randomized clinical trials have reported significant long-term beneficial symptom-related effects of radon balneotherapy in rheumatic diseases, lasting up to nine months post-intervention. Compared to radon-free treatments, radon balneotherapy was superior in terms of pain relief, function improvement, reduction in anti-inflammatory and analgesic drug consumption, and persistence of these benefits over a longer term [126,127,128]. Moreover, a meta-analysis by Falkenbach and co-workers [129] showed significantly better pain reduction in the long term after radon spa therapy in rheumatic pathologies.

Overall, the results suggest beneficial long-term clinical effects of radon spa therapy—consistent with the concept of hormesis—as a complementary strategy in the treatment of rheumatic conditions, especially RA and OA.

Table 1 presents a summary of the most relevant studies—according to quality (original research, appropriate experimental design and methodology, English language, indexed in PubMed) and originality—regarding potential biological biomarkers mediating clinical benefits of different modalities of spa therapy, and proposed hormetic mechanisms participating, at least partially, in these effects.

## 6. Conclusions

Balneotherapy is an effective complementary approach in the management of several low-grade inflammation- and stress-related pathologies, especially rheumatic and metabolic conditions. However, despite the demonstrated clinical and symptomatic benefits of these therapies, their role in modern medicine is still controversial, mainly because the biological mechanisms underlying these benefits have not yet been completely elucidated. In the context of these pathologies, further studies are clearly necessary in order to clarify the mechanisms of effectiveness involving the stress response and, consequently, its interaction with the inflammatory response.

In this review, we proposed that neuroendocrine and immune effects are very important biological mechanisms of effectiveness of this therapy, and that several hormetic pathways can be involved in these effects. Due to the variety and heterogeneity of balneotherapy modalities, water and mud compositions, and application protocols, it is difficult to determine the exact intervention for obtaining optimal biological and clinical outcomes in different pathologies. Furthermore, the regulation of altered inflammatory and stress status by this strategy could be conditioned by each specific disease’s basal set-point, so whether the benefits of balneotherapy could be extended to other conditions or even healthy subjects remains unknown.

In the context of hormesis, it is necessary to ascertain the ideal temperature and concentration of different bioactive chemical elements (as well as the number and duration of sessions, and intervals between each session) in order to elicit hormetic responses without causing damaging or toxic effects. Further studies looking deeper into the hormetic mechanisms of effectiveness are clearly needed, so balneotherapy can be practiced by health professionals based on scientific evidence that supports its use.

## Figures and Tables

**Table 1:** Potential hormetic mechanisms and biomarkers mediating the clinical benefits of different modalities of spa therapy.

Study	Treatment(s)	Main Hormetic Mechanism	Experimental Subjects	Pathology	Biomarkers	Clinical Benefits
Yamashita et al., 1998 [86]	Hydrotherapy (40–42 °C, 1 session of 5–15 min)	Heat stress	Rats	Myocardial ischemia	Increased Hsp72 and manganese-SOD myocardial levels	Biphasic reduction in the incidence of ventricular fibrillation and in the size of the myocardial infarction
Okada et al., 2004 [87]	Hydrotherapy (41 °C, 28 daily sessions of 15 min)	Heat stress	Rats	Inflammatory arterial lesions	Reduced monocyte/macrophage infiltration and MCP-1 expression in the adventitia of arteries; increased expression of Hsp72 in the adventitia and media of arteries	Suppression of neointimal thickening
Bathaie et al. 2010 [88]	Hydrotherapy (42 °C, 60 daily sessions of 30 min)	Heat stress	Rats	Diabetes	Serum HDL increased whereas LDL, TG, and TC decreased; insulin and eHsp72 serum levels increased; AGE products serum levels decreased; serum antioxidant capacity improved	Prevention of diabetes complications and increased survival
Kavanagh et al. 2016 [89]	Hydrotherapy (40 °C, 10 daily sessions of 30 min)	Heat stress	Monkeys	Insulin resistance	Increased muscle Hsp70 levels; reduction in plasma glucose concentration; improved insulin secretion and normalized responses to glucose challenge	Improved blood pressure and glucose metabolism
Hooper 1999 [85]	Hydrotherapy (38–41 °C, 18 daily sessions of 30 min)	Heat stress	Humans	Type 2 diabetes mellitus	Fasting plasma glucose and glycated hemoglobin levels decreased	Body weight decreased and glucose metabolism improved
Ortega et al. 2017 [49]	Balneotherapy, using water rich in bicarbonate and calcium, and mud (38–42 °C, 10 daily sessions of 60 min)	Heat stress	Humans	Osteoarthritis	Levels of serum inflammatory cytokines (IL-1β, TNF-α, IL-8, IL-6, and TGF-β) decreased; cortisol serum levels increased and eHsp72 serum levels decreased	Pain reduction; improved knee flexion angle, stiffness and physical function; better health-related quality of life
Uzunoğlu et al. 2017 [91]	Balneotherapy, using water rich in bicarbonate and calcium (39–40 °C, 21 daily sessions of 15 min)	Heat stress	Humans	Osteoarthritis	Initial and transient increase in serum eHsp72 and IFN-γ levels after first session, but final decrease of these biomarkers at the end of the protocol	Not evaluated
Benedetti et al. 2010 [45]	Balneotherapy using sulfurous water at 37 °C and mud at 46–48 °C (12 daily sessions of 20 min); with (Group A) or without (Group B) drinking 400 mL of the water daily	Hydrogen sulfide	Humans	Osteoarthritis	Group A: increase in plasma thiol levels, decrease in plasma MDA and carbonyl levels, and in serum TNF-α and COMP levels; all of them at the end of the treatment and at 1-month follow-up. Plasma MMP-2 levels decreased only at the end of the treatment.Group B: plasma MDA and carbonyl levels, and serum TNF-α levels decreased only at the end of the therapy	Pain reduction
Benedetti et al. 2009 [114]	Balneotherapy consisting of drinking sulfurous water (500 mL daily for 2 weeks)	Hydrogen sulfide	Humans	Healthy	Decreased plasma MDA, carbonyls, and advanced oxidation protein products levels; increased plasma antioxidant capacity and thiol levels	Not evaluated
El-Seweidy et al. 2011 [115]	Balneotherapy consisting of drinking sulfurous water (ad libitum daily for 7 weeks)	Hydrogen sulfide	Rats	Diabetes	Serum concentrations of insulin, C-peptide and IGF-1 increased; glycemia and glycated hemoglobin levels decreased.Cardiac GSH and thiol levels increased; glutathione disulfide levels decreased; reduction in NF-κβ, MMP-2, TGF-β1, procollagen-1 and Fas-L gene expression in the left ventricle	Prevention of the development of diabetes-induced fibrosis in the heart: normal myocytes and absence of collagen
Sadik et al. 2011 [116]	Balneotherapy consisting of drinking sulfurous water (ad libitum daily for 7 weeks)	Hydrogen sulfide	Rats	Diabetes	Serum concentrations of insulin, C-peptide and IGF-1 increased; glycemia and glycated hemoglobin levels decreased.Testosterone serum levels and testicular GSH increased; testicular overexpression of Bax/Bcl-2, cytochrome *c*, caspase-9 and -3, and p53 was blocked.	Prevention of diabetes-induced testicular dysfunction: improved seminiferous tubule structure, number of spermatogenic cells and hormonal function
Safar et al. 2015 [117]	Balneotherapy consisting of drinking sulfurous water (ad libitum daily for 6 weeks)	Hydrogen sulfide	Rats	Diabetes	Glycemia and glycated hemoglobin levels decreased.Decreased creatinine and urea serum levels; decreased renal thiobarbituric acid reactive substances levels; increased renal GSH levels	Prevention of diabetes-induced nephropathy: improved kidney function and absence of histopathological alterations
Yamaoka et al. 2004 [121]	Spa therapy consisting of inhalating radon at 36 °C (Group A), or sauna bath at 48 °C in the absence of radon (Group B) (5 sessions of 40 min)	Radon	Humans	Healthy	Group A and B: SOD and catalase activity, and insulin and glucose-6-phosphate dehydrogenase levels increased; lipid peroxide levels and total cholesterol decreased.Group A only: decreased percentage of CD8^+^ cells and increased percentage of CD4^+^ cells. Increased α-atrial natriuretic polypeptide levels, ACTH, and β-endorphins; decreased vasopressin levels.	Not evaluated

ACTH: adrenocorticotropic hormone; AGE: advanced glycation end; COMP: cartilage oligomeric protein; eHsp: extracellular heat shock protein; GSH: glutathione; HDL: high-density lipoprotein; Hsp: heat shock protein; IFN-γ: interferon gamma; LDL: low-density lipoprotein; MCP-1: monocyte chemoattractant protein-1; MDA: malondialdehyde; MMP: matrix metalloproteinases; NF-κβ: nuclear factor kappa beta; SOD: superoxide dismutase; TC: total cholesterol; TG: triglycerides.

## Abbreviations

ACTH	Adrenocorticotropic hormone
AGE	Advanced glycation end
Akt	Protein kinase B
AMPK	AMP-activated protein kinase
COMP	Cartilage oligomeric matrix protein
CRP	C-reactive protein
eHsp	Extracellular heat shock protein
FM	Fibromyalgia
GH	Growth hormone
GSH	Glutathione
H_2_S	Hydrogen sulfide
HDL	High-density lipoprotein
HPA	Hypothalamic-pituitary-adrenal
HS	Heat shock
IFN-γ	Interferon gamma
iHsp	Intracellular heat shock protein
LDL	Low-density lipoprotein
LTB4	Leukotriene B4
MCP-1	Monocyte chemoattractant protein-1
MDA	Malondialdehyde
MMP	Matrix metalloproteinases
NA	Noradrenaline
NF-κβ	Nuclear factor kappa beta
NO	Nitric oxide
OA	Osteoarthritis
OARSI	Osteoarthritis Research Society International
PBMC	Peripheral blood mononuclear cells
PGE2	Prostaglandin E2
RA	Rheumatoid arthritis
RANTES	Regulated on Activation, Normal T-cell Expressed and Secreted
RNS	Reactive nitrogen species
ROS	Reactive oxygen species
SNS	Sympathetic nervous system
SOD	Superoxide dismutase
TC	Total cholesterol
TG	Triglycerides
